# Influence of probiotics on water quality in intensified *Litopenaeus vannamei* ponds under minimum-water exchange

**DOI:** 10.1186/s13568-022-01370-5

**Published:** 2022-02-26

**Authors:** Marwa A. Hassan, Mustafa A. Fathallah, Mohamed A. Elzoghby, Mohamed G. Salem, Mohamed S. Helmy

**Affiliations:** 1 Department of Animal Hygiene, Zoonoses and Behavior, Faculty of Veterinary Medicine, Suez Canal University, Ismailia, 41522 Egypt; 2 Fish Farming and Technology Institute, Suez Canal University, Ismailia, 41522 Egypt

**Keywords:** Water probiotics, Water quality, Unionized ammonia, Magnetic field

## Abstract

The effects of two probiotics on NH_3_ degradation, as well as the magnetic field (21.56 m tesla) on the germination and proliferation of *Bacillus* spores, were studied in-vitro. Additionally, the effect of these probiotics on water quality maintenance in *Litopenaeus vannamei* holding ponds was investigated. For 180 min, NH_3_ degradation was assessed as follows: Set 1: ammonia-free tap water with NH_3_; Probiotic A (5 × 10^10^ viable *Bacillus* spores/g) with NH_3_; Probiotic B (multi spp. 2 × 10^9^ CFU/g) with NH_3_; and Set 2: same as set 1 with 30 mg L^−1^ OM. The magnetic field was tested on Probiotic A (3.5 × 10^7^ CFU) for 36 h in triplicate. In the presence of organic matter, both probiotics degrade NH_3_. The viable Bacillus count increased within 6 h of being exposed to the magnetic field, reaching its peak after 36 h. Firstly, fifteen ponds (250,000 PL/acre) were investigated, then 360 water samples were collected from the same corresponding pond for 8 weeks, and subjected to T1: control; T2: Probiotic A (0.007 g/m^3^/2 weeks); T3: Probiotic B (0.03 g/m^3^/2 weeks). Both probiotics with TVC and NH_3_ demonstrated a negative correlation, on the other hand, they showed a significant (P ≤ 0.01) improvement in DO and pH. Overall, both probiotics were able to degrade NH_3_ and the magnetic field (21.56 m tesla) was efficient to improve the germination and proliferation of *Bacillus* spores in-vitro. Probiotics were also effective for reducing TVC and NH_3_ levels by increasing dissolved oxygen and pH in pond water.

## Introduction

Aquaculture is a major source of food and nutrition for millions of people globally, and it is essential to address the world’s critical food demand (Low et al. 2017). As a result, intensive aquaculture to meet the demands of a rapidly growing population has been laden with challenges (Edwards 2015). Consequently, high stocking densities cause rapid deterioration of water quality, leading to stress and increased susceptibility to diseases as it is a suitable environment for the proliferation of pathogenic microbes, and, eventually, the mortality of cultured species (Lieke et al. 2019; Zokaeifar et al. 2014). Instantaneously, increased organic carbon, suspended particles, phosphates, nitrogenous species (nitrates, nitrites, and ammonia), chemical oxygen demand, and biological oxygen demand are frequently related to aquaculture effluent. Generally, water pollution in aquaculture is unavoidable since aquatic organism culture is accompanied by waste accumulation, which pollutes receiving waters and groundwater, moreover, aquaculture effluents are discharged into natural water bodies (Lalloo et al. 2007).

Water exchange and biofiltration are traditional approaches for controlling hazardous metabolites in aquaculture (Jahangiri and Esteban 2018), also several systems and methods for improving water quality and treating aquaculture wastewater have also been proposed and implemented which include recirculating aquaculture systems, biofloc technology, and aquaponics (Emerenciano et al. 2017; Maucieri et al. 2018; Rijn 2013). The most common approach for maintaining aquaculture water quality is frequent water exchange, which is costly, time-consuming, and may introduce pathogens into culture systems (Devaraja et al. 2013). Likewise, water scarcity and low water quality are becoming an international issue, particularly in arid and semi-arid regions; hence, the use of low-quality irrigation water is gaining importance in many countries around the world’s agricultural sector (Aleman et al. 2014). Various researches are being conducted to investigate the efficacy of probiotics in the management of aquaculture water quality (Hura et al. 2018). Probiotics, in this context, can play a vital role in aquaculture productivity by improving non-specific disease prevention and providing pollution-free water sources (Panigrahi et al. 2010). Probiotics have been frequently proposed as environmentally friendly replacements to antibiotics (Jahangiri and Esteban 2018). Pond probiotics’ antibacterial activity is mediated by variables such as the synthesis of bacteriocins, siderophores, lysozyme, protease, hydrogen peroxide, pH alterations, and the formation of organic acids and ammonia (Verschuere et al. 2000). Furthermore, in aquaculture systems, probiotics function through diverse mechanisms to eliminate organic wastes and contaminants because of the combination of ‘bioremediation’ and ‘biocontrol’ when dealing with environmental concerns. According to some findings, when probiotics were administered through the water compared to other administration protocols, a high level of integration of probiotic bacteria into treated aquatic organisms (especially in marine environments) was observed, possibly due to continuous drinking in the aquatic environment (Olafsen 2001; Villamil et al. 2010). The practical application and use of magnetic field treatments in agriculture have a wide range of applications, including seed germination, seedling development, and yields of various species, and poultry production, which plays an important role in addressing the shortage of nutrition in developing countries (Khalil et al. 2016). Similarly, magnetic treatment was revealed to enhance germination in *Bacillus megaterium*, *Bacillus cereus*, and *Bacillus subtilis* spores, and these spores’ magnetic properties may have biotechnological applications, particularly in detection and separation (Zhou et al. 2018). Although the use of probiotics as water additives in ponds is thought to be more appropriate because it can be performed on all stages of fish (Jahangiri and Esteban 2018), Few studies have been conducted to investigate their use as water additives in fish production systems (Kord et al. 2022). There are few publications on the efficacy of probiotics and their comprehensive mechanism of action. Most researches are conducted in a laboratory setting; thus, the potentiality may differ when these probiotics are applied in natural settings (ponds and lakes) (Hasan and Banerjee 2020).

As a result, the current study was conducted to investigate the effects of two different probiotics used as water additives on NH_3_ degradation as well as the effect of a magnetic field on the germination and proliferation of *Bacillus* spores probiotic was studied in vitro. Furthermore, the effect of those probiotics on water quality in earthen ponds with Whiteleg shrimp (*Litopenaeus vannamei*) and a minimum-water exchange system.

## Materials and methods

### Probiotics composition

To conduct the study, the two most used probiotics in the field of aquaculture in Egypt were evaluated both in vitro and in pond treatment. Probiotic A (Sanolife PRO-W^®^, INVE aquaculture Company, Thailand) is a single species probiotic of a total 5 × 10^10^ viable spores per gram (*B. subtilis*, 2.75 × 10^10^ CFU/g and *B. licheniformi*, 2.25 × 10^10^ CFU/g); the recommended dose is 0.007 g/m^3^/2 weeks (Total of 3.5 × 10^8^ CFU/g/m^3^ containing: *B. subtilis*, 1.93 × 10^8^ CFU/g and *B. licheniformi*, 1.58 × 10^8^ CFU/g). While probiotic B (Aquastar^®^, Biomin, Austria) is a mixture of probiotics consisting of Multi species with a total of 2 × 10^9^ CFU/g (*Bacillus *spp., 5 × 10^8^ CFU/g; *Pediococcus* spp., 1.25–1.5 × 10^9^ CFU/g; and *Enterococcus* spp., 5 × 10^7^ CFU/g); the recommended dose is 0.03 g/m^3^/2 weeks (Total of 6 × 10^7^ CFU/g/m^3^ containing: *Bacillus* spp., 1.5 × 10^7^ CFU/g; *Pediococcus* spp., 4 × 10^7^ CFU/g; and *Enterococcus* spp., 1.5 × 10^6^ CFU/g).

### In vitro evaluation of probiotics on NH_3_ degradation

In vitro trial for the effectiveness of both probiotics on reduction of NH_3_ was conducted at 25 ºC by dissolving ammonium chloride (APHA 2017) in ammonia-free tap water (10 L); pH 7.9 ± 0.1 with a final concentration of 1 mg N^−1^ and 1.22 mg NH_3_ L^−1^ into 2 sets, each designed in triplicates, the first set includes 3 treatments: T1; control (ammonia-free tap water with NH_3_); T2 (Probiotic A with NH_3_) and T3; (probiotic B with NH_3_); while the second set included T4; T5 and T6, which were designed just like the first set in addition to the presence of organic matter (30 mg L^−1^ OM), which was selected based on a previously conducted survey (data are not shown). Yeast suspension (Lab018, United Kingdom) was added as a source of organic matter and was measured according to Kumar (1992). NH_3_ levels were measured at 30; 60; 120 and 180 min after initial addition.

### In vitro evaluation of magnetic field on the propagation of *Bacillus* spores probiotic

Single species probiotic was selected for easy culturing and counting. Evaluation of magnetic field on viable *Bacillus* spores of the probiotic was conducted on 2 systems of water tanks at 25 ºC, the first system served as control system supplied with aerators, that consisted of 3 tanks (1000 L capacity) and the second system without aerators and consisted of 3 tanks (1000 L capacity) with magnetic field system (magnet with a whole field strength of 21.56 m tesla divided into 10.78 m tesla in each side; control unite which disconnected the field every 3 h. work for one hour rest; and water motor). A single spp. probiotic 0.7 g (3.5 × 10^10^ CFU) was dissolved in 1 L sterile saline (0.85%) then 100 mL was added to each sterilized perchlorinated water tank (100 L) to final dose of (3.5 × 10^7^ CFU). Water samples for viable spore bacillus count were collected after 3, 6, 12, 24, and 36 h post-application on BHI agar for 24 h. at 30 ºC using drop plate technique (Herigstad et al. 2001)**.**

### Evaluated ponds conditions

This study was conducted to evaluate the efficiency of 2 types of probiotics in maintaining water quality in whiteleg shrimp (*Litopenaeus vannamei*) farm in East Delta, Egypt. This study was conducted on a farm with a total pond area of 3900 m^2^/earthen pond filled with marine water. The water depth of about 1.8 m between June and August 2019. The post-larvae of white shrimp (*L. vannamei*) were obtained from a private hatchery in June. Ponds were dried for one month and the topsoil was scraped and removed, plowed, and filled with pre-chlorinated (12 ppm available chlorine) water from a reservoir pond. Aerators were deployed in each pond according to the stocking density of the pond at the rate of one paddlewheel aerator (1 HP motor) for 1-hectare post larvae (PL).

Each pond was stocked with whiteleg shrimp (*L. vannamei*) at the rate of 250,000 PL/acre with an average initial weight 0.005 g. A good quality high protein (38%) commercial feed from Skretting Egypt Company (Table 1) was provided 3 times a day as per the feeding schedule given by the feed manufacturer.

**Table 1:** Proximate composition (% dry matter) of the diet

Items	%
Moisture	7.45
Crude protein	37.92
lipid	6.5
Crude Ash	9.7
NFE	45.88
Gross energy	440.65

Based on NRC (1993) for protein, lipid and carbohydrate respectively

Productivity was based on the natural productivity of the ponds hence the experimental ponds were kept free from any shading throughout the day. A total of 15 ponds were used in this investigation, which were examined for water parameters before the conduction of the study (n = 45 water samples), after that, a total of 360 water samples (n = 120 sample/5 ponds/treatment) were collected from the same corresponding pond during the study period of 8 weeks and the ponds were classified into 3 treatments (5 pond/treatment); T1: the control ponds; T2: ponds treated with single spp. probiotic; T3: ponds treated with multi spp. probiotic.

### In pond probiotics evaluation on water quality

Two different probiotic brands were added to ponds’ water every 2 weeks, besides the control ponds with no probiotic. In the control ponds (T1) water exchange rate was one-third of the pond’s water every 3 days, while in the other treated ponds the following strategy was applied: in T2: water in ponds was not exchanged for 7 days after the first use (50 g/ pond) of the probiotic (2 days before PL stocking and 5 days after), every 2 weeks the probiotic was applied into the ponds which were closed for 5 consecutive days, in T3 the ponds were closed for 10 days after application (200 g/ pond) of the probiotic (5 days before PL stocking and 5 days after), then water exchange rate in each treatment was applied every 2 weeks as one-third of the pond’s water. Sampling for physicochemical parameters was collected from specific points of the pond at a depth of 50 cm below the surface. Temperature, pH using (Jenway, 370-pH meter, U.K) and DO (mg L^−1^) by dissolved oxygen meter (DO-meter; Crison OXI 45 P, EU), salinity (g L^−1^), by conductivity meter (Jenway, 4520 conductivity meter, UK) were measured onsite daily, according to the standard procedures and methods as defined in APHA (2017). OM (mg L^−1^) according to (Kumar 1992), and toxic ammonia (NH_3_) (mg L^−1^) were analyzed spectrophotometrically at weekly intervals (Thermo Spectra, USA) following standard procedures (APHA 2017). Water samples for the total *Vibrio* count (TVC) were collected weekly and serially diluted and plated on TCBS medium (Hi-media, Mumbai) for 24–48 h at 30 °C using the drop plate technique described by (Herigstad et al. 2001).

### Statistical analysis

The data were statistically analyzed using SPSS version 22 computer program (Inc., 1989–2013), results were expressed as means ± SE for each treatment and were subjected to one way ANOVA analysis of variance LSD test performed to test the significant difference between the treatments at *P* ≤ 0.01and *P* ≤ 0.05. The Pearson correlation coefficient was performed as a correlation matrix in the form of a rectangular array of numbers, which gives the correlation coefficient of the variables with each other. Factor analysis was performed as principal components analysis (PCA) according to the method described by Liu et al. (2003), to summarize the major relationship between variables by showing distribution and variation within the physicochemical, KMO and Bartlett’s sphericity test were used to verify the applicability of PCA to raw data (Zhang et al. 2020). Then, the corresponding correlation coefficient matrix, and eigenvalues were calculated. The principle that the eigenvalue > 1.0 to determine the number of principal components was accepted (Mandal et al. 2008). The maximum variance was used to normalize the rotation of the original data to rotate the factor using Varimax with Kaiser normalization (Kaiser 1958).

## Results

### Effect of probiotics on NH_3_ (mg L^−1^)

**Fig. 1:**
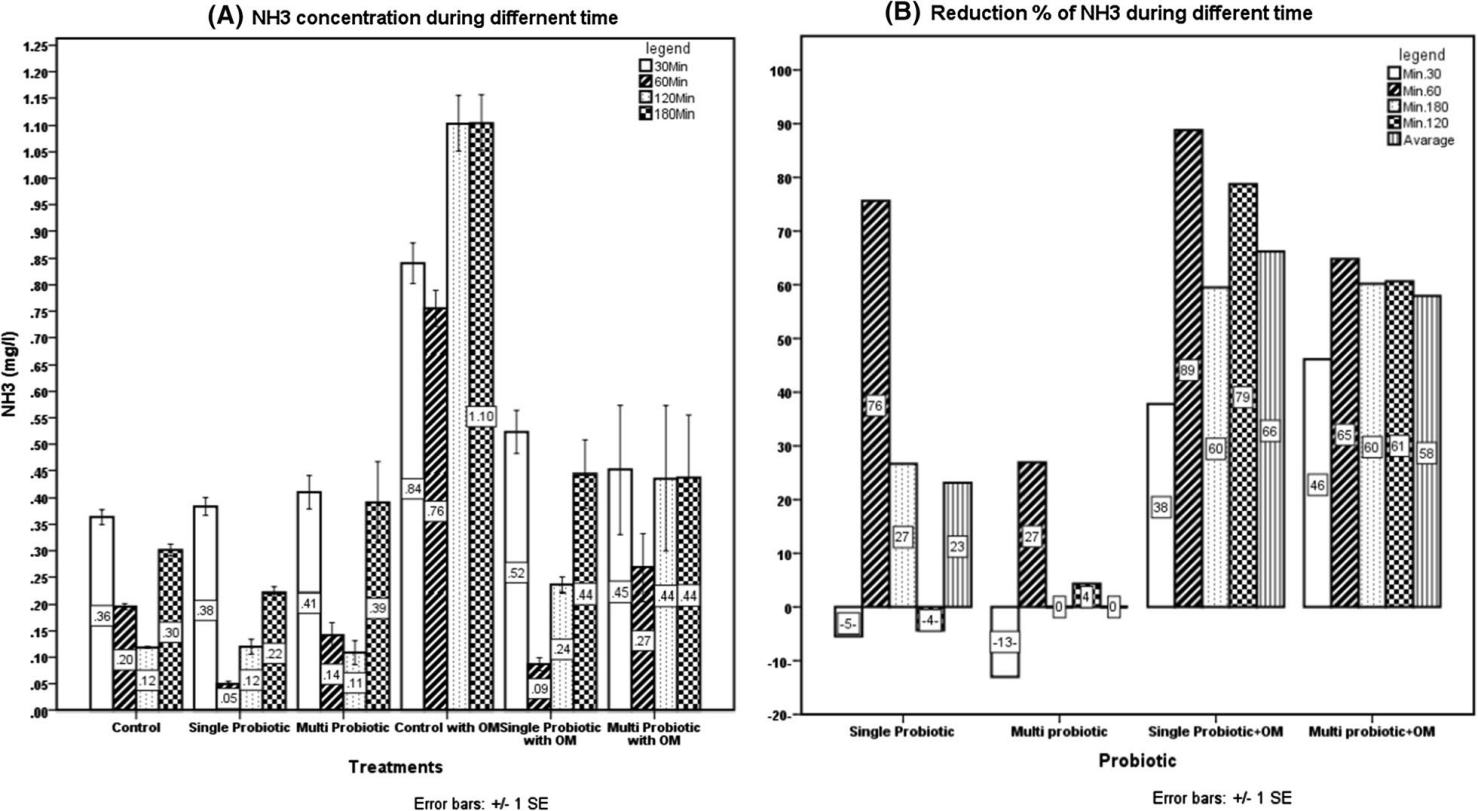
Effect of probiotics on unionized ammonia (NH_3_) level in the presence or absence of organic matter. Means are statistically different (P ≤ 0.01)

Treatment with single spp. probiotic positively enhanced the significant (P ≤ 0.01) reduction in NH_3_ level during the different experimental time compared to control with higher reduction percentage; the effect of multi spp. probiotic was shown as a trend toward a decrease in the level of NH_3_ when compared to the control group. Regarding the effect in the presence of OM, both probiotics showed a significant decrease in NH_3_ level (P ≤ 0.01) compared to control; maximum reduction effect (P ≤ 0.01) in single spp. probiotic was pronounced than the mixture of probiotics within 60 and 120 min (Fig. 1).

### Effect of magnetic field on *Bacillus* spores probiotic

**Fig. 2:**
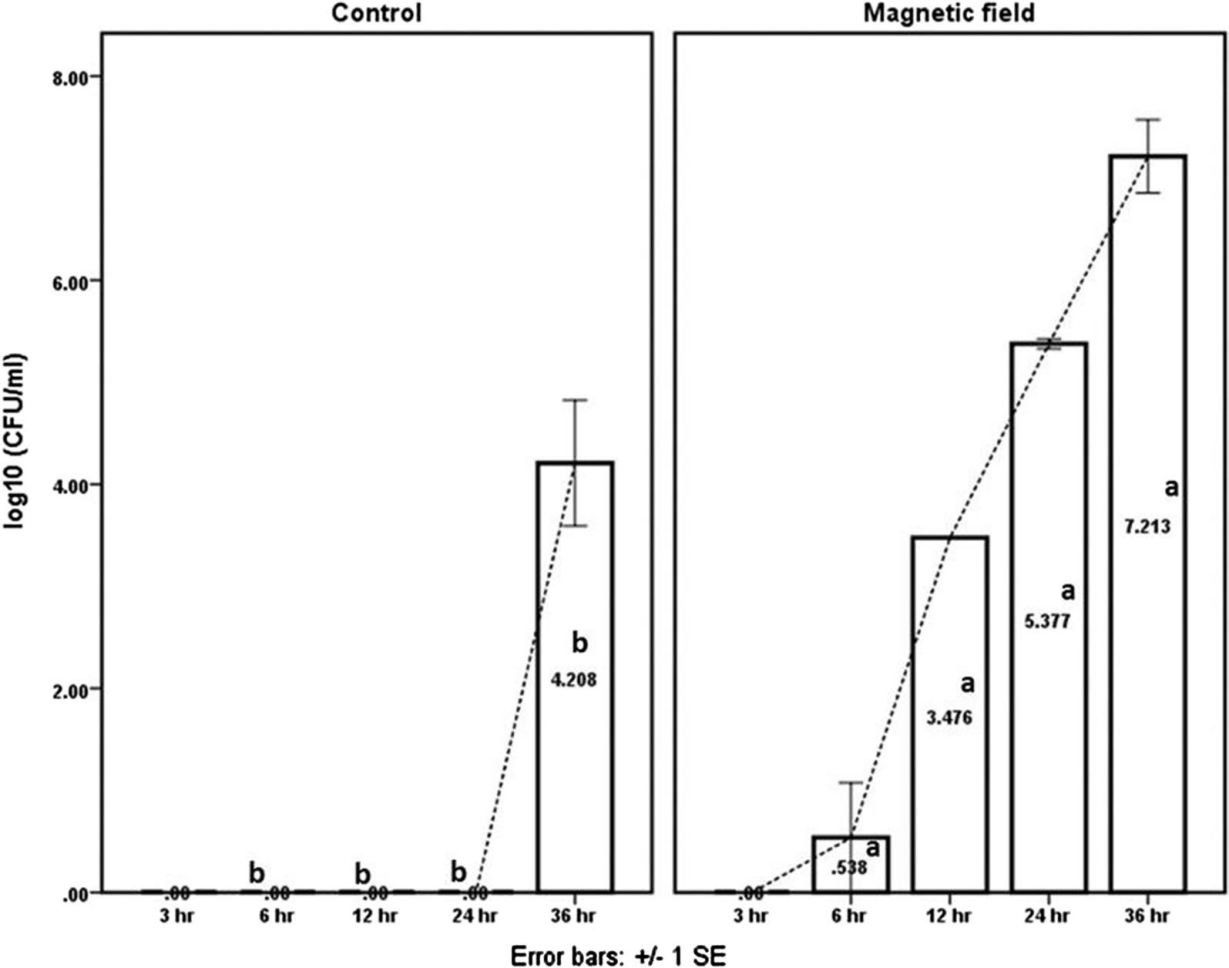
Effect of magnetic field on *Bacillus* spore’s germination and propagation. Means are different letters are significantly differing at P ≤ 0.0001

The effect of magnetic field (21.56 m Tesla) on *Bacillus* spores was evaluated and data is illustrated in Fig. 2, the first detection of the viable cell count in magnetic field treatment was recorded 6 h after application and the count was gradually elevated with the increasing time, one the other hand, after 36 h *Bacillus* were detected in the control system with significantly (P ≤ 0.0001) lower count compared to magnetic field application.

### Total ***Vibrio*** count (log_10_ CFU mL^−1^) of water pond

A significant (P ≤ 0.01) decrease in TVC (log_10_ CFU mL^−1^) in both T2 and T3 was observed before and after treatment as well as when compared to control ponds (Table 2). TVC in control ponds (T1) correlated positively with temperature; pH and NH_3_ and negatively with salinity (Table 3). Both probiotics and TVC were found to have a significant (P ≤ 0.01) negative correlation (Table 3).

**Table 2:** Some physicochemical parameters of the examined fish water ponds before and after application of probiotic

Treatments	Control ponds (T1)		Single spp. probiotic (T2)		Multi spp. probiotic (T3)		Permissible limits according to	
Application to the pond	Before stocking	After stocking	Before stocking	After stocking	Before stocking	After stocking		
Total *Vibrio* count (log CFU mL^−1^)*	3.764^b^ ± 0.129	3.63^b^ ± 0.137	4.408^a^ ± 0.173	1.263^c^ ± 0.193	4.055^ab^ ± 0.213	0.856^c^ ± 0.188		
Temperature (ºC)*	29.019^a^ ± 0.123	29.271^a^ ± 0.229	28.621^a^ ± 0.182	29.068^a^ ± 0.1	28.734^a^ ± 0.13	29.068^a^ ± 0.095	24–30	(Clifford 1997; Lucas and Southgate 2012)
Dissolved oxygen (m gL^−1^)	4.127^d^ ± 0.078	4.782^c^ ± 0.102	4.141^d^ ± 0.077	5.965^b^ ± 0.431	4.603^ cd^ ± 0.017	6.541^a^ ± 0.294	> 4	Lucas and Southgate (2012)
pH	7.614^b^ ± 0.059	7.554^bc^ ± 0.071	7.903^a^ ± 0.026	6.995^d^ ± 0.08	7.917^a^ ± 0.017	7.314^c^ ± 0.056	6.5–9.5	Carbajal-Hernández et al. (2012)
Salinity (g L^−1^)	35.355^ab^ ± 1.499	37.590^a^ ± 1.452	31.690^ab^ ± 0302	34.0^ab^ ± 0.533	30.483^b^ ± 0.128	32.136^ab^ ± 0.519	15–45	(Clifford 1997; Lucas and Southgate 2012)
Organic matter (m gL^−1^)	15.479^b^ ± 0.216	16.298^ab^ ± 0.194	16.103^b^ ± 0.229	12.75^c^ ± 0.665	16.828^a^ ± 0.141	15.636^b^ ± 0.626		
NH_3_ (m gL^−1^)	0.049^a^ ± 0.003	0.026^b^ ± 0.001	0.050^a^ ± 0.004	0.006^c^ ± 0.001	0.030^b^ ± 0.002	0.005^c^ ± 0.0001	0.01–0.09	Boyd and Zimmermann (2000)

The means with different superscript letters within the same row for each parameter are significantly different at *P ≤ 0.01 and at P ≤ 0.05

**Table 3:** Correlations coefficient between different water quality parameters in the examined pond with or without probiotic

Parameters		Total *Vibrio* log (CFU mL^−1^)	Temperature (ºC)	Dissolved oxygen (mg L^−1^)	pH	Salinity (g L^−1^)	Organic matter (mg L^−1^)	NH_3_ (mg L^−1^)
	Probiotics	− 0.622**	**0.074**	0.536**	− 0.452**	**0.008**	− 0.451**	− 0.472**
Total *Vibrio* log (CFU mL^−1^)	− 0.688**	1	**0.132**	− 0.348**	0.537**	− 0.324**	0.269**	0.511**
Temperature (ºC)	0.040	0.127	1	**− 0.066**	**0.028**	**0.031**	− 0.384**	**0.021**
Dissolved oxygen (mg L^−1^)	0.708**	− 0.480**	− 0.057	1	− 0.354**	0.304**	− 0.373**	− 0.604**
pH	− 0.289**	0.472**	0.025	− 0.180*	1	− 0.584**	0.301**	0.527**
Salinity (g L^−1^)	− 0.049	− 0.262**	0.034	0.253**	− 0.612**	1	**− 0.070**	− 0.549**
Organic matter (mg L^−1^)	− 0.037	0.116	− 0.447**	0.154*	0.098	− 0.070	1	**0.129**
NH_3_ (mg L^−1^)	− 0.498**	0.554**	0.032	− 0.659**	0.476**	− 0.511**	− 0.086	1
Control (non treated ponds)								
Total *Vibrio* log (CFU mL^−1^)		1	0.255**	− 0.112	0.424**	− 0.431**	0.055	0.315**
Temperature (ºC)			1	− 0.056	0.039	0.031	− 0.488**	0.062
Dissolved oxygen (mg L^−1^)				1	0.033	0.545**	0.048	− 0.651**
pH					1	− 0.677**	0.062	0.405**
Salinity (g L^−1^)						1	− 0.085	− 0.621-**
Organic matter (mg L^−1^)							1	− 0.156
NH_3_ (mg L^−1^)							− 0.156	1

Probiotics: upper diagonal represents single spp. and lower one represents multi spp.

*Correlation is significant at the 0.05 level (2-tailed)

**Correlation is significant at the 0.01 level (2-tailed)

### Water pond temperature (ºC) and dissolved oxygen (mg L^−1^)

Water temperature (ºC) showed almost the same pattern before and after the treatment with non-significant change among the treatments (Table 2).

Although the levels of dissolved oxygen (DO) mg L^−1^ found a non-significant difference in all ponds before the application of probiotics to the water, the highest level of DO was recorded in T3 followed by T2 which was significantly different from the control ponds (Table 2). A significant (P ≤ 0.01) positive relationship was observed between the level of DO in water with T3 and T2, (Table 3).

### Water pond salinity (g L^−1^) and pH

Regarding the water salinity (g L^−1^), a non-significant difference with a trend toward an increase in the treated ponds after treatment was recorded (Table 2). The pH of the ponds after treatment showed a significant decrease (P ≤ 0.05) than that of the pretreatment, in addition, a significant (P ≤ 0.05) decrease in the pH values was recorded in each of the ponds treated with probiotics, while T2 recorded the lowest significant pH (P ≤ 0.05) between treatments (Table 2). The pH value showed a strong negative correlation (P ≤ 0.01) with both T2 and T3 (Table 3).

### Organic matter (mg L^−1^) and Toxic ammonia NH_3_ (mg L^−1^)

A significant decrease (P ≤ 0.05) was reported in organic matter (OM) mg L^−1^ in the post-treated ponds with T2 and T3 than pre-treated ponds, and T2 also recorded the lowest values among the treatments (Table 2). NH_3_ levels behaved the same pattern as well as pH values in pre- and post-treated ponds, as well as in response to probiotics application (Table 2). A highly significant (P ≤ 0.01) inverse relationship between probiotic inT2 and OM has been reported (Table 3). A negative correlation (P ≤ 0.01) was observed between the use of both probiotics and NH_3_ (Table 3).

### Principal component analysis of water quality parameters

Principal components analysis (PCA) was conducted as it is an effective tool to figure out the driving factors of water quality with probiotics effect in a deep sight. The parameters in all treatments produced three principal components (PC) explaining the total variances of 79.296%, 75.457, and 77.092% for T1; T2, and T3 respectively. Corresponding, variable loadings and explained variance are presented in Table 4. PC1 in T1 had positive loadings (> 0.75) on DO and significant negative loadings with NH_3_; PC2 was characterized by strong positive loadings of TVC, and pH; weak loading with NH_3_ and negative loading with salinity; PC3 showed a high positive loading of temperature. Regarding the effect of probiotics, PC1 in T2 was saturated mainly by high and moderate negative loads of probiotic and DO, respectively; on the other hand, high positive loading of TVC; and moderate for NH_3_ and weak for pH and OM was recorded. PC1 in T3 is mainly affected by a high positive load of probiotic; DO and high negative loads with TVC; NH_3_ and pH.

**Table 4:** Principal component analysis of the treatments with water quality parameters

Treatments	Control (non-treated ponds)			Single spp. probiotic			Multi spp. probiotic		
Component	1	2	3	1	2	3	1	2	3
Probiotic effect				**− 0.912**	**− 0.004**	0.163	**0.949**	**0.058**	− 0.091
Total *Vibrio* log (CFU mL^−1^)	− 0.054	**0.778**	0.149	**0.727**	**0.321**	0.112	**− 0.755**	**0.316**	0.04
Temperature (ºC)	0.044	0.179	0.872	0.148	− 0.036	0.911	− 0.016	0.041	− 0.836
Dissolved oxygen (mg L^−1^)	**0.941**	0.082	− 0.033	− 0.681	− 0.298	0.065	**0.842**	**− 0.123**	0.143
pH	− 0.145	**0.874**	− 0.078	**0.448**	**0.704**	− 0.058	**− 0.242**	**0.828**	0.056
Salinity (g L^−1^)	0.663	− 0.635	0.142	**0.014**	**− 0.954**	0.038	**0.023**	**− 0.922**	− 0.021
Organic matter (mg L^−1^)	0.104	0.13	− 0.852	0.493	0.004	− 0.718	0.021	0.086	0.866
NH_3_ (mg L^−1^)	**− 0.813**	0.386	0.117	**0.541**	**0.678**	0.056	**− 0.663**	**0.575**	− 0.107
Initial Eigenvalues	2.024	1.976	1.55	2.582	2.059	1.395	2.68	1.993	1.494
% of variance	28.921	28.234	22.141	32.279	25.736	17.441	33.497	24.918	18.677
Cumulative %	28.921	57.155	79.296	32.279	58.016	75.457	33.497	58.415	77.092

### Discussion

In vitro evaluation of the capability of the used probiotics in NH_3_ degradation was conducted either with or without OM; the result revealed that both probiotics reduced NH_3_ in the presence of high OM (30 mg L^−1^) which simulates the pond conditions. And this result proves the ability of probiotics in degradation of NH_3_ in presence of OM, meanwhile, multi spp. probiotics take more time due to the various composed microorganisms. According to Mahardhika et al. (2019), they indicated that *Lactobacillus* spp. and *Bacillus* spp. can inhibit the chemical reaction of the ammonia formation between uric acid and water and the uricase enzyme from gram-negative bacteria, such as most bacteria that conduct the nitrifying process originated from *Nitrobacter*, *Nitrosomonas*, and *Nitrococcus*. Additionally, the toxic ammonia had been reported to be degraded by *Bacillus substili*s (Cha et al. 2013), *Bacillus amyloliquefaciens* (Xie et al. 2013), *Bacillus coagulans*, and *Lactobacillus plantarum* (Mi et al. 2019). Moreover, Anwar et al. (2021) conducted a study to screen bacteria having a capacity to degrade ammonia as NH_4_Cl in vitro using 5 bacterial strains (*IBP-1*, *IBP-2*, *IBP-3*, *IBP-4*, and *IBP-5* strains) and they found that the five bacterial isolates were able to degrade the ammonia content.

In natural conditions, probiotics should be introduced to the pond 2–7 days before aquatic animal stocking for activation, which varies depending on the type of probiotics spp.; to shorten this period, single spp. (*Bacillus*) probiotics were chosen to investigate the prospective influence of magnetic field on spore germination. Magnetic field exposure increased the number of viable *Bacillus* count within 6 h, with the maximum effect at 36 h. One possible explanation for the magnetic field effect is that it promotes germination by enhancing their environment as a physical mean of treatment and regulating the quorum sensing process. Spore germination is typically stimulated by (i) nutrition such as sugars, purine nucleosides, and amino acids, (ii) non-nutrient agents such as CaDPA, surfactants, or dodecylamine, or (iii) physical treatments (Paidhungat et al. 2001; Setlow 2014). Germination, which is primarily characterized by spore rehydration and resistance loss, is only the very first stage in the process leading to the initiation of the first cell division and the establishment of a daughter population (Bressuire-Isoard et al. 2018). Xu Zhou et al. (2018) observed that *Bacillus* spores are paramagnetic because of the high manganese content accumulated within the spore core and that linked with the sport coat; and the intrinsic magnetic strength of the spore was adequate to facilitate their separation using relatively strong magnetic fields. Furthermore, *Bacillus* germination is a highly coordinated process that is controlled by quorum sensing and a cell–cell communication mechanism (Liu et al. 2016). Bacteria can count their numbers and regulate their activities by detecting the signaling molecules that are generated when the concentration of the signaling molecules exceeds certain thresholds (Bassler and Losick 2006). According to Zhang et al. (2011), communication between endospores occurs during germination, with closer endospores eliciting more synchronized behavior.

The mechanism by which *Bacillus* spore germination could be improved, as well as the hatchability of Artemia as a live feed of shrimp, as magnetic fields can affect membrane functions, not only by a local effect on ion fluxes or ligand binding but also by altering the distribution and aggregation of intramembranous proteins (Bersani et al. 1997).

Microorganisms in intensive aquaculture play a key role in influencing productivity, nutrient cycling, disease outbreaks, and environmental protection (Moriarty and Decamp 2005). *Vibrio* is a common bacteria found in a variety of aquatic and marine ecosystems; of the more than 100 *Vibrio* species discovered, approximately 12 types can cause human infections, while others cause diseases in marine animals (Huang et al. 2021). In general, dissolved organic carbon (DOC) has been shown to have a significant impact on *Vibrio* ecology (Takemura et al. 2014). Furthermore, *Vibrio* spp. are obligate heterotrophs that depend on organic matter for carbon sources and use a variety of them for nutrition; additionally, *Vibrio* spp. may integrate, consume, and produce organic matter, altering its chemical characteristics and bioavailability (Zhang et al. 2018). In the current study, both probiotics significantly reduced TVC, with a negative correlation, and this finding could be supported by research published by Kord et al. (2022), who indicated that the use of multiple probiotic species as water additives in Tilapia production may combat TVC abundance and prevent the occurrence of fish diseases. Simultaneously, (Xu Zhou et al. 2018) discovered that utilizing Bacillus spp. probiotics in a water pond reduced the presence of *Vibrio harveyi*, *Vibrio vulnificus*, *Vibrio parahemolyticus*, and *Vibrio vulnificus*.

The most reasonable explanation is that probiotics actively assimilate or break down organic matter or toxic material, hence improving environmental quality (Hemaiswarya and Doble 2013). Gram-positive *Bacillus* spp. convert organic matter back to CO_2_ more efficiently than gram-negative bacteria, which convert a higher amount of organic carbon to bacterial biomass or slime (Stanier et al. 1963), gram-positive bacteria in the production pond can decrease dissolved and particulate organic carbon buildup during the culture cycle while enhancing more stable phytoplankton blooms through increased CO_2_ production (Scura 1995). At the same time, probiotics synthesize more enzymes than natural bacteria, including amylase, protease, lipase, xylanase, and cellulase, which aid in waste decomposition (Araujo et al. 2016; Banerjee et al. 2017; Giri et al. 2013). The water quality parameters indicated that alkaline pH conditions, as well as elevated temperature and ammonia levels, favor the emergence of high *Vibrio* counts (Heenatigala and Fernando 2016; Hsieh et al. 2008). Furthermore, TVC had a proper relationship with pH, NH_3_ and temperature in control ponds, but this relationship was inverted with the addition of probiotics, which improved water quality while making it unfavorable for *Vibrio* proliferation. Douillet (1998) observed that a probiotic additive composed of a mixture of bacteria in a liquid suspension improved water quality in fish and crustacean cultures by lowering the concentration of OM and ammonia, which was accomplished by a series of enzymatic processes carried out in sequence by the different strains present in the probiotic mixture, as a result, the addition of this blend improved water quality.

The physicochemical parameters of pond water are vital determinants of water quality and a culture system’s ability to support fish production (Kord et al. 2022). The levels of all water physicochemical parameters obtained in this study were within the acceptable limits for shrimp culture (Boyd and Zimmermann 2000; Carbajal-Hernández et al. 2012; Clifford 1997; Lucas and Southgate 2012; Reddy and Mounika 2018).

The data obtained showed no variation in water temperature among treatments, which is consistent with the findings of several authors (Banerjee et al. 2010; Ghosh et al. 2008; Nimrat et al. 2012) who found no significant impact of Bacillus species on temperature. According to Velmurugan and Rajagopal (2009), the temperature may be unaffected by a biological activity because it is a conservative measure.

DO particularly is a very critical water quality parameter that supports all living things including fish (Kord et al. 2022). DO levels in the present study were significantly enhanced by the different probiotics microorganism species used as water additives. A few studies have found that Bacillus probiotic can keep DO levels in the optimal range. Higher DO values were obtained in an experiment conducted by Hura et al. (2018) who investigate the influence of *Bacillus* *megaterium* on water quality in the cultivation of major carps. A blend of *Bacillus* species improved DO levels in an aquarium filled with tilapia larvae at high density (Hainfellner et al. 2018). Higher DO was documented in *Bacillus* supplemented waters during transport of fish (Yellowfin Tuna Yolk Sac Larvae and Carnegiella strigata) in additional tests utilizing *Bacillus* species (*B. subtilis*, *Bacillus licheniformis*, *B. megaterium*, *and Bacillus laterosporous*) (Gomes et al. 2008; Zink et al. 2011). Nevertheless, no significant difference in DO levels was seen when a mixture of *B. megaterium* and *Streptomyces fradiae* was used for water treatment (Kord et al. 2022). It may be argued that research on the modulation of DO by probiotic *Bacillus* is less investigated when compared to its effects on nitrogenous species in aquaculture (Hlordzi et al. 2020). Furthermore, Nitrifies, Sulphur bacteria, *Bacillus* spp., *Pseudomonas* spp., *Bacillus* spp., *Bacillus toyoi*, *Streptomyces* (Das et al. 2008); *Lactobacillus plantarum*, *Lactobacillus casei* (Melgar Valdes et al. 2013) as water probiotics could enhance dissolve oxygen concentration.

The current study proposes that both probiotics added to the ponds’ water correlated negatively with pH, resulting in a significant reduction in its value, which was not online with the findings of Kord et al. (2022), who observed that different probiotics added to the Nile *Tilapia* ponds’ water did not affect the pH value. The concentration of hydrogen ions (pH) and alkalinity have an impact on practically every biological and chemical process, making them critical water quality factors (Summerfelt et al. 2015).

Considering, the effect of water probiotics on salinity modulation was not statistically significant. Because salinity is a conservative metric for water quality, biological processes have a challenging time modifying it (Velmurugan and Rajagopal 2009). *Bacillus* species have no significant effect on salinity as a water quality indicator, according to the limited study that has been conducted. Salinity, for example, was unaffected by *B. pumilus* (Banerjee et al. 2010; Sreenivasulu et al. 2016) or a mixture of *B. megaterium* and *Streptomyces fradiae* (Aftabuddin et al. 2013; Banerjee et al. 2010).

The current investigation revealed that the *Bacillus* spp. probiotic (T2) had the lowest OM concentration with a significant negative correlation between them. Gram-positive genus *Bacillus* group is more efficient than gram-negative genus in converting organic matter to CO_2_, it is suggested that maintaining high levels of probiotics in production ponds will reduce the accumulation of dissolved and particulate organic carbon during the growing season (Raja 2015), in addition, this can balance phytoplankton production (Balcázar et al. 2006). Previous research found that Bacillus spp. reduced organic matter loads in treated ponds compared to controls, resulting in higher water quality (Moriarty and Decamp 2005). Bacterial species belonging to the genera *Bacillus*, *Pseudomonas*, *Nitrosomonas*, *Nitrobacter*, *Acinetobacter*, and *Cellulomonas* are known to help in the mineralization of organic water and in reducing the accumulation of organic loads (Shariff et al. 2001).

In this study, the use of both probiotics resulted in a significant reduction in water pH and, as a result, NH_3_ levels, and this association was confirmed by the strong negative correlation between probiotics and both pH and NH_3_. This decrease in NH_3_ levels compared to the control group is consistent with those obtained by Kord et al. (2022), who reported a significant decrease in NH_3_ with the application of different probiotics into Nile *Tilapia* water ponds, and attributed this to the enhanced microbial activity that absorbed the nitrogenous compounds and used them in their metabolism. Furthermore, introducing probiotics to pond water may have enhanced the population of nitrifying bacteria, allowing ammonia to be converted to nitrite and subsequently to nitrate. This was consistent with the findings of (Wang et al. 2005), who discovered that adding probiotics to shrimp ponds significantly increased the population of nitrifying bacteria *Nitrosomonas* and *Nitrobacter*. Ammonia levels in shrimp water ponds can be reduced by introducing probiotics, according to (Wang 2007). The addition of the following probiotics resulted in NH_3_ reduction: *Bacillus* species (*Bacillus subtilis*, *Bacillus licheniformis*, *Bacillus megaterium*, *and Bacillus laterosporous*) (Gomes et al. 2008; Zink et al. 2011); *Lactobacillus acidophilus* (Porubcan 1991); *Bacillus* NL110 (Mujeeb Rahiman et al. 2010); Lactobacillus acidophilus (Khademzade et al. 2020; Porubcan 1991) discovered that adding *Pediococcus acidilactici* and *Bacillus cereus* bacteria to fishponds significantly reduced nitrogenous particles. (El-Kady et al. 2021) found also that commercial probiotics used as water additives reduced NH_3_ and total ammonia–nitrogen as compared to the control treatment.

Generally, water temperature, pH, dissolved oxygen, and NH_3_ were improved in trials with probiotics, and daily administration of probiotics demonstrated the ability to maintain a healthy environment for shrimp and prawn larvae in the improved green water system (Banerjee et al. 2010).

In conclusion, the use of probiotics (single spp. or multi spp.) as water additives with minimum water exchange improved water quality by lowering TVC and NH_3_ levels and increasing DO levels. In-vitro testing of the ability of the applied probiotics in NH_3_ degradation demonstrated that both probiotics could reduce NH_3_ in the presence of high OM (30 mg L^−1^), simulating pond conditions. Furthermore, in-vitro investigation of the influence of magnetic field on spore germination and proliferation revealed that magnetic field exposure increased the number of *Bacillus* spores within 6 h, with the maximum effect at 36 h, which will reduce the time required for activation of *Bacillus* spores’ probiotics before stocking of PL. Further studies are needed to evaluate different magnetic fields strengths in aquaculture.

## Data Availability

The authors declare that they do not have any shared data available.
